# Mild-to-moderate renal pelvis dilatation identified during pregnancy and hospital admissions in childhood: An electronic birth cohort study in Wales, UK

**DOI:** 10.1371/journal.pmed.1002859

**Published:** 2019-07-30

**Authors:** Lisa Hurt, Melissa Wright, Joanne Demmler, Judith VanDerVoort, Susan Morris, Fiona Brook, David Tucker, Maria Chapman, Nick A. Francis, Rhian Daniel, David Fone, Sinead Brophy, Shantini Paranjothy

**Affiliations:** 1 Division of Population Medicine, Cardiff University School of Medicine, Cardiff, United Kingdom; 2 Centre for Trials Research, Cardiff University, Cardiff, United Kingdom; 3 Swansea University Medical School, Swansea, United Kingdom; 4 Cardiff and Vale University Health Board, University Hospital of Wales, Cardiff, United Kingdom; 5 Aneurin Bevan University Health Board, Caerleon, Newport, United Kingdom; 6 Congenital Anomaly Register and Information Service, Singleton Hospital, Swansea, United Kingdom; 7 Antenatal Screening Wales, Public Health Wales, Cardiff, United Kingdom; University of Manchester, UNITED KINGDOM

## Abstract

**Background:**

Chronic kidney disease (CKD) is a growing contributor to the global burden of noncommunicable diseases. Early diagnosis and treatment can reduce the severity of kidney damage and the need for dialysis or transplantation. It is not known whether mild-to-moderate renal pelvis dilatation (RPD) identified at 18–20 weeks gestation is an early indicator of renal pathology. The aim of this follow-up to the Welsh Study of Mothers and Babies was to assess the risk of hospital admission in children with mild-to-moderate antenatal RPD compared with children without this finding. We also examined how the natural history of the RPD (whether the dilatation persists in later pregnancy or postpartum) or its characteristics (unilateral versus bilateral) changed the risk of hospital admission.

**Methods/Findings:**

This population-based cohort study included singleton babies born in Wales between January 1, 2009, and December 31, 2011 (*n* = 22,045). We linked ultrasound scan data to routinely available data on hospital admissions from the Patient Episode Database for Wales (PEDW). The outcome was a hospital admission for urinary tract causes (defined by an expert study steering group) in the first three years of life. We used Cox regression to model time to first hospital admission, according to whether there was evidence of RPD at the fetal anomaly scan (FAS) and/or evidence of dilatation in later investigations, adjusting for other predictors of admission. We used multiple imputation with chained equations to impute values for missing data. We included 21,239 children in the analysis. The risk of at least one hospital admission was seven times greater in those with RPD (*n* = 138) compared with those without (*n* = 21,101, conditional hazard ratio [cHR] 7.23, 95% confidence interval [CI] 4.31–12.15, *p* < 0.001). The risk of hospital admission was higher in children with RPD at the FAS and later dilatation (cHR 25.13, 95% CI 13.26–47.64, *p* < 0.001) and in children without RPD at the FAS who had later dilatation (cHR 62.06, 95% CI 41.10–93.71, *p* < 0.001) than in children without RPD (*n* = 21,057). Among children with RPD at the FAS but no dilatation in later pregnancy or postpartum, we did not find an association with hospital admissions (cHR 2.16, 95% CI 0.69–6.75, *p* = 0.185), except when the initial dilatation was bilateral (cHR 4.77, 95% CI 1.17–19.47, *p* = 0.029). Limitations of the study include small numbers in subgroups (meaning that these results should be interpreted with caution), that less severe outcomes (such as urinary tract infections [UTIs] managed in the community or in outpatients) could not be included in our analysis, and that obtaining records of radiological investigations later in pregnancy and postpartum was challenging. Our conclusions were consistent after conducting sensitivity analyses to account for some of these limitations.

**Conclusions:**

In this large population-based study, children with RPD at the FAS had higher rates of hospital admissions when there was persistent dilatation in later pregnancy or postpartum. Our results can be used to improve counselling of parents and develop care pathways for antenatal screening programmes, including protocols for reporting and further investigation of RPD.

## Abstract 2

Shantini Paranjothy and coworkers investigate whether renal pelvis dilatation identified during the midpregnancy ultrasound scan is associated with higher hospital admission rates during childhood.

## Author summary

**Why was this study done?:**

- Pregnant women usually have an ultrasound scan when they are about 20 weeks pregnant. The purpose of this anomaly scan is to look for structural abnormalities, such as abnormal growth or development of organs.
- It is not known if some of the findings at this scan are indicators of health problems.
- For example, fluid-filled areas in the baby’s kidneys (called ‘renal pelvis dilatation’) are sometimes seen, but it is not known whether these are associated with adverse outcomes in childhood.

**What did the researchers do and find?:**

- In this study, children with renal pelvis dilatation, and a group of children who did not have this finding, were followed until they were three years old.
- Babies who had this dilatation were more likely to be admitted to hospital with problems in the kidneys and urinary tract than children without dilatation, especially if the finding was still present later in pregnancy or after the birth (HR 25.13, 95% CI 13.26–47.64, *p* < 0.001). In children with dilatation at the anomaly scan, but no dilatation in later pregnancy or postpartum, we did not find an association with hospital admissions (HR 2.16, 95% CI 0.69–6.75, *p* = 0.185), except when the initial dilatation was in both kidneys (HR 4.77, 95% CI 1.17–19.47, *p* = 0.029).

**What do these findings mean?:**

- These results can be used to improve counselling of parents and to develop care pathways for antenatal screening programmes, including protocols for reporting and further investigation of renal pelvis dilatation.

## Introduction

Chronic kidney disease (CKD) is a growing contributor to the global burden of noncommunicable diseases [1,2]. Although relatively rare in children [1], management of CKD in paediatric patients is complex and costly [1,2], and the condition has a profound impact on children and their families [3]. Approximately half of all cases of CKD in children in high income countries are due to congenital anomalies of the kidney and urinary tract (CAKUT) [4,5]. Early diagnosis and treatment can reduce the severity of kidney damage and the need for dialysis or transplantation [6,7].

Routine investigations during pregnancy are an opportunity to screen for CAKUT. In the United Kingdom, all women are offered an ultrasound scan at 18 to 20 weeks gestation to detect major anomalies in the fetus. The National Health Service (NHS)’s Fetal Anomaly Screening Programme specifies that 11 structural abnormalities are detectable at this scan, including one CAKUT (bilateral renal agenesis) [8], but other abnormalities of the kidney and urinary tract can also be identified [4,7]. One such finding is dilatation of the fetal renal pelvis (the collecting system where urine flows from the kidney into the ureter), detected by measuring the anterior-posterior (AP) diameter of the renal pelvis. This is also known as hydronephrosis, pelvicalyceal dilatation, pelviectasis, or pyelectasis [9]. Current UK guidance recommends that this finding should be reported and assessed further [8,10]. However, different classification systems exist, which use different criteria and nomenclature to distinguish between potential pathological dilatation and transient changes that are of limited clinical significance [6,11,12], leading to inconsistent management and parental anxiety [13,14].

Measurement thresholds based on ‘best available evidence…[of] prognostic information’ have been proposed [6], with AP dilatation in the second trimester of 4- to <7-mm classified as mild, 7- to ≤10-mm as moderate, and >10-mm as severe. Third trimester AP thresholds of 7- to <9-mm are classified as mild, 9- to ≤15-mm as moderate, and >15-mm as severe. Severe dilatation is reported to be associated with postnatal pathology in almost 90% of cases [15]. However, previous studies examining the sequelae of mild or moderate antenatal dilatation have found conflicting results, have several methodological limitations, and have been assessed to be of low or moderate quality [15–21]. These have not therefore led to the development of consistent care pathways [12,22,23].

The Welsh Study of Mothers and Babies is a prospective, population-based cohort that was established to examine childhood morbidity associated with ultrasound findings of unknown significance detected at the fetal anomaly scan (FAS). We analysed data from this cohort with the aim of assessing the risk of hospital admission in the first three years of life associated with mild-to-moderate antenatal renal pelvis dilatation (RPD). We also examined how the natural history of the RPD (whether the dilatation persists in later pregnancy or postpartum) or its characteristics (unilateral versus bilateral) changed the risk of hospital admission.

## Methods

The Welsh Study of Mothers and Babies recruited a cohort of pregnant women receiving antenatal care in Wales between 2008 and 2011, to estimate the prevalence of seven nonstructural findings at the FAS and examine their association with pregnancy outcomes and longer-term health outcomes [24]. Ethical approval for the original study was given by the Multicentre Research Ethics Committee for Wales (reference 08/MRE09/17) on April 16, 2008.

### Study population

All pregnant women who had a second trimester FAS in six of seven Welsh Health Boards between July 2008 and March 2011 were eligible for inclusion. At recruitment, women were asked to give written consent that the data from their ultrasound scan could be linked with routinely collected data on their child.

### Inclusion and exclusion criteria

**Fig 1:**
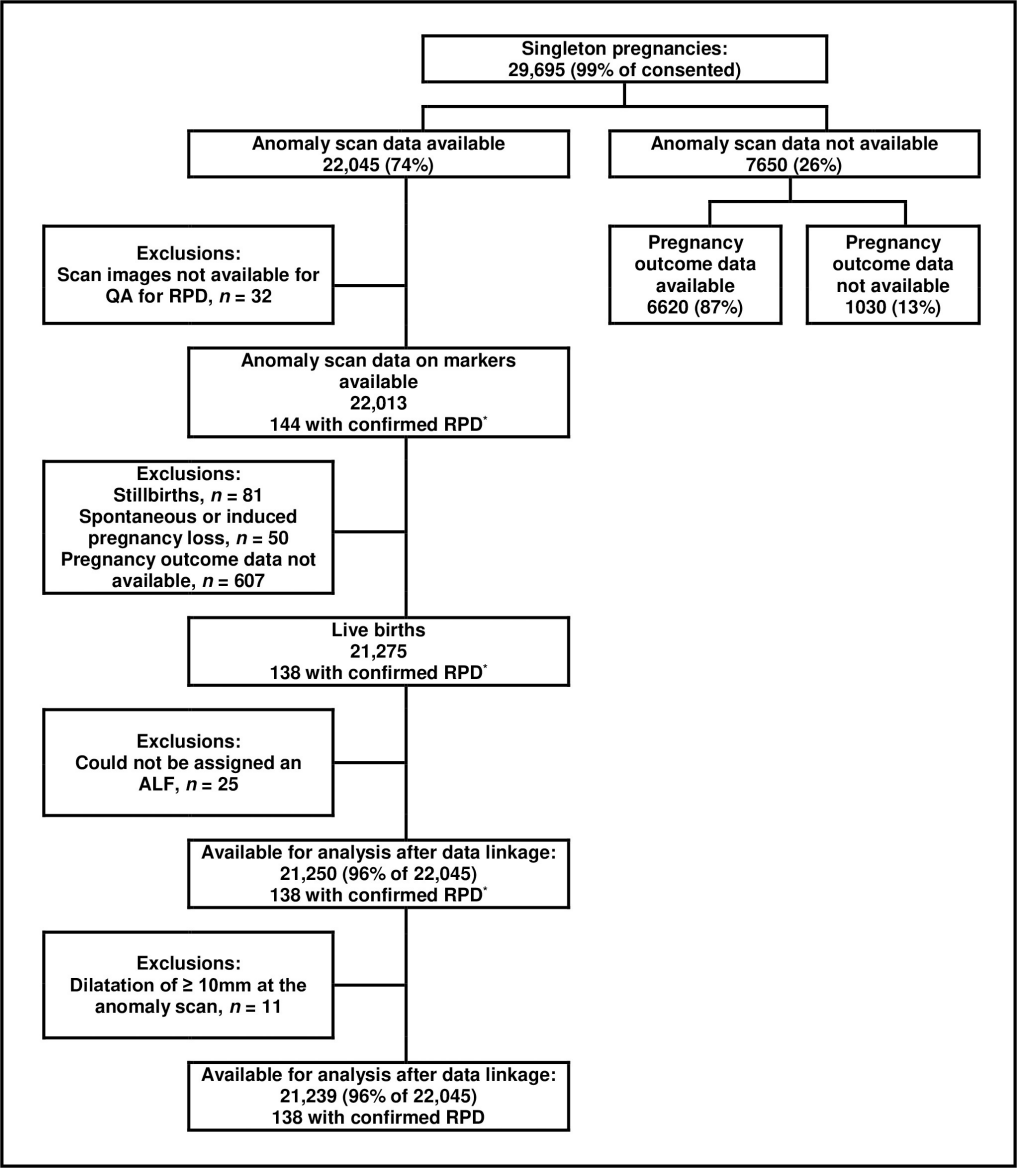
Cohort flow diagram. ALF, anonymised linking field; QA, quality assurance; RPD, renal pelvis dilatation.

The population for this analysis was singleton children who were live-born between January 1, 2009, and December 31, 2011, to mothers in Wales who consented to take part in the study and for whom validated FAS data were available (Fig 1). Pregnancies with an unknown outcome (for example, because the birth happened outside of Wales) were excluded. Children whose information could not be assigned an anonymised linking field (ALF; for example, because they did not receive their healthcare in Wales or did not have a valid NHS number or other identification variables) were also excluded, as linkage with the healthcare datasets was not possible. Children were followed from birth until the occurrence of death, migration out of Wales, 3rd birthday, or December 31, 2014 (end of follow-up). Person-time was censored in cases of death and migration.

### Definition of exposure

RPD was defined as fluid-filled dilatation of the renal pelvis measured on the axial section, with an AP diameter of 5.0 to 9.9 mm (with the callipers to be placed on the inner AP margins of the renal pelvic wall) at the FAS [25]. An additional reporting screen was added to the information system for radiological data storage and reporting in Wales (Radiology Information Service 2 [RadIS2]) to capture the scan data. Data were also collected on whether the dilatation was unilateral or bilateral. An expert quality assurance (QA) panel reviewed FAS images to confirm the presence of dilatation in accordance with the study definition; the number of cases of RPD reduced from 221 to 144 after this process (for detail, see [26]).

**Fig 2:**
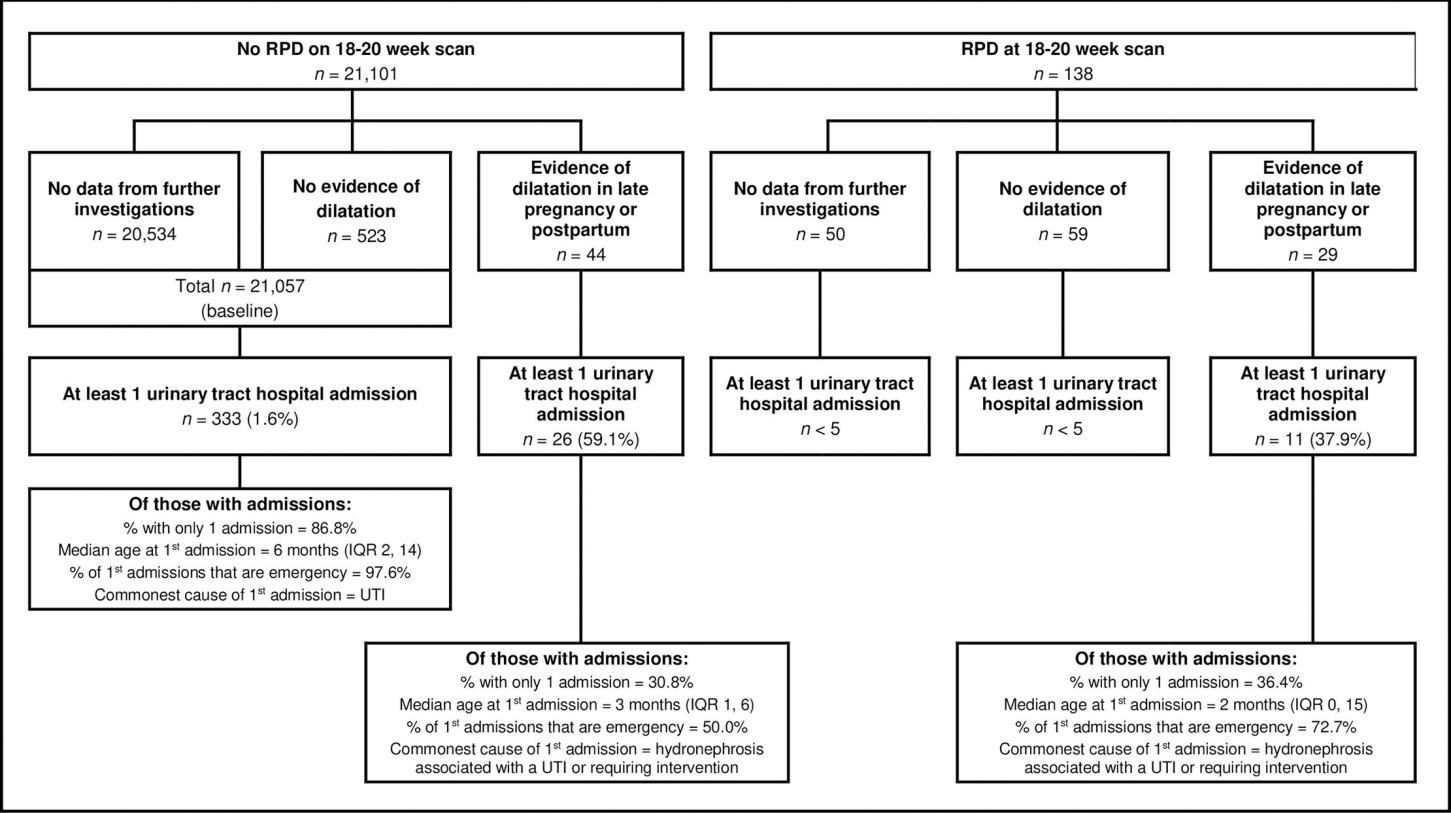
Definition of exposure groups for the analysis, and characteristics of hospital admissions. IQR, interquartile range; RPD, renal pelvis dilatation; UTI, urinary tract infection.

We also sought to obtain information from radiological investigations conducted later in pregnancy from RadIS2 and the Congenital Anomaly Register for Wales (CARIS). Data on the presence of dilatation identified later in pregnancy and/or up to 12 months postpartum, plus any measurements recorded, were extracted from radiological reports. In accordance with international guidance [6,12], measurements of 7.1 mm or greater later in pregnancy or postpartum were considered evidence of later dilatation. The exposure groups in the analysis were then stratified into (i) no RPD at FAS or later, (ii) no RPD at FAS but RPD present later, (iii) RPD at FAS but not later, and (iv) RPD at FAS and later (Fig 2).

### Definition of outcomes

The outcome was a hospital admission for urinary tract causes in the first three years of life. An admission was defined as a continuous stay using a hospital bed provided by the NHS in Wales under one or more consultants, and included transfers between hospitals. A list of codes for possible causes of admissions in children for which RPD could be considered a possible precursor (including renal and lower urinary tract problems) was agreed by the study steering group, which included a consultant paediatric nephrologist, consultant radiologists, and an academic general practitioner (see S1 Table). The list was based on the International Statistical Classification of Diseases and Related Health Problems, 10th Revision (ICD-10) [27] and the procedure code list used in the NHS (Operating Procedure Codes Classification of Interventions and Procedures version 4 or OPCS-4) [28]. Hospital admissions with any of these codes in any coding position were identified from the Patient Episode Database for Wales (PEDW). Admissions as a day case for postnatal investigations alone are not a part of this dataset, and these admissions would not therefore have been included.

### Data linkage

Data from the Welsh Study of Mothers and Babies were exported to the Secure Anonymised Information Linkage (SAIL) Databank [29] to enable the radiological data to be linked with data on hospital admissions (from PEDW), congenital anomalies (from CARIS), deaths (from the Office for National Statistics Annual District Death Extract), and migration (from the Welsh Demographic Service data). For each of these datasets, individuals were assigned a unique identifier (the ALF) provided by the NHS Wales Informatics Service. The linkage system uses a combination of deterministic (based on NHS numbers) and probabilistic record linkage (based on first name, surname, date of birth, gender, and phonex and soundex versions of names); this linkage is more than 99.85% accurate [30]. Second-stage encryption is used by the data bank before storing data, and third-stage encryption is used to create project-specific linked datasets. Approval from the Information Governance Review Panel of the SAIL Databank was obtained for the analysis.

### Sample size

Preliminary analyses of PEDW data showed that 2 per 100 children were admitted to hospital with a urinary tract cause before the age of five years. We therefore estimated that we would need approximately 21,000 children without RPD and 140 children with RPD to detect, at a 5% type 1 error rate, a 3-fold increase in urinary tract hospital admissions with RPD with 80% power. There were enough children in our cohort for this analysis.

### Statistical methods

The statistical analysis was planned by the investigators of the study, in collaboration with the study steering group, in advance of the conduct of the analysis (for detail, see [24] and the protocol for this analysis, S1 Renal Study Protocol). This included the specification of the ICD-10 codes to be included in the definition of the outcome. An initial analysis of the association between RPD and hospitalisations was presented to the study steering group in 2016, and they recommended that the additional radiological data (from later pregnancy and postpartum) were sought and added to the analyses. We used Cox regression to model time to the first urinary tract hospital admission to three years of age. Our primary outcome was time to first hospital admission because most children with an admission were only admitted once (81.3%). We estimated hazard ratios (HRs) with 95% confidence intervals (CIs) to examine the risk of hospital admissions associated with the presence of RPD at the FAS, and then according to whether the child had RPD and/or later dilatation. The proportional hazards assumption was assessed graphically using log-minus-log plots and was tested based on the Schoenfeld residuals. We originally planned to include follow-up time until the child’s fifth birthday, but there was evidence that the proportional hazards assumption was violated in this analysis because all of the admissions in the RPD group occurred before the age of three years, whereas children without RPD continued to be admitted for the first time after three years of age. Based on this and on peer review comments, we chose a cutoff of three years of age for the follow-up period (instead of the preplanned five years), and formal testing confirmed there was no strong evidence that the proportional hazards assumption was violated in these models (for example, *p* = 0.11 for the binary RPD variable). Conclusions from models including five years of follow-up were similar to the models including three years of follow-up.

We examined associations in unadjusted models and conditional on other predictors of hospital admissions (sex, maternal age in three categories [<25, 25–34, 35+ years], deprivation quintile based on the UK Townsend Deprivation Score [31], and prematurity). We repeated the analyses to estimate HRs stratified according to whether the RPD was unilateral or bilateral. We also conducted sensitivity analyses: (i) adding information on dilatation identified during hospital admissions into the definition of exposure subgroups (that is, also using codes Q62.0, N13.0, N13.1, N13.2, or N13.3 in any position for a PEDW admission as evidence of later dilatation) and (ii) using an Anderson-Gill model to account for multiple urinary tract admissions during the follow-up period.

Data from radiological investigations to assess for dilatation later in pregnancy or postpartum were missing for 50 of the 138 children with RPD. There was also a low percentage of children with missing data on covariates (0.7% for Townsend score, 0.3% for prematurity). Multiple imputation with chained equations [32] was used to impute values for the missing data (10 imputations) under the missing at random assumption. The imputation model included all covariates, the outcome variable (urinary tract admissions), and the cumulative baseline hazard [33]. Conclusions from a complete case analysis and from the multiple imputation were similar. In response to peer-review comments, we present the results from the multiply imputed datasets.

All analyses were conducted within the SAIL Gateway using Stata version 15.1.

The study is reported as per the Strengthening the Reporting of Observational Studies in Epidemiology (STROBE) guideline (see S1 STROBE Checklist).

## Results

Anomaly scan data were available for 22,045 children (Fig 1). The characteristics and pregnancy outcomes of their mothers were comparable to the general population of pregnant women in Wales [26]. Thirty-two pregnancies were excluded, as their scan images were not available for QA for RPD. There were 81 stillbirths, 50 spontaneous or induced abortions, 607 pregnancies with no outcome data, and 25 babies who could not be assigned an anonymised linkage field identifier, leaving 21,250 children available for analysis after data linkage. Eleven children had dilatation of the renal pelvis measuring 10.0 mm or greater at the FAS. All of these had evidence of a urinary tract hospital admission or significant renal pathology in investigations after birth and were excluded from all further analyses. This analysis is therefore based on 21,239 children (96.3% of those with scan data) who contributed 61,984 child-years of follow-up.

A total of 138 children (0.7%) had confirmed RPD at the FAS (Table 1). RPD was more prevalent in male than female children (0.9% compared with 0.4%) and in children of younger mothers (0.9% when maternal age was <25), but there was no association with area-level social deprivation, prematurity, or low birth weight. Overall, 1.8% of the children had at least one admission for a urinary tract problem during follow-up (Table 2). Girls were more likely than boys to have at least one admission, as were children of younger mothers and premature children.

**Table 1:** Characteristics of the cohort, by RPD status at anomaly scan.

Characteristics	RPD at anomaly scan	
	No	Yes
	*N* = 21,101 (99.3%)	*N* = 138 (0.7%)
	Number (%)	Number (%)
**Sex**		
Male	10,804 (99.1)	95 (0.9)
Female	10,297 (99.6)	43 (0.4)
**Maternal age**		
<25	6,158 (99.1)	53 (0.9)
25–34	11,694 (99.5)	62 (0.5)
35+	3,249 (99.3)	23 (0.7)
Townsend score*		
1	3,529 (99.4)	20 (0.6)
2	3,334 (99.4)	20 (0.6)
3	4,087 (99.4)	25 (0.6)
4	4,779 (99.4)	30 (0.6)
5	5,218 (99.2)	43 (0.8)
Premature**		
No	19,921 (99.3)	131 (0.7)
Yes	1,125 (99.4)	7 (0.6)
**Birth weight**^†^		
≥2,500 g	19,882 (99.3)	131 (0.7)
< 2,500	1,090 (99.4)	7 (0.6)

*Townsend deprivation score: 1 = least deprived, 5 = most deprived; missing data for 154 (0.7%).

**Prematurity: premature = <37 weeks gestation; missing data for 55 (0.3%).

^†^Missing data for 129 (0.6%); all later analyses include prematurity but not birth weight, as these two variables are highly correlated; prematurity has fewer missing values, and the effect of the two variables in the multivariate models was similar.

Abbreviation: RPD, renal pelvis dilatation.

**Table 2:** Characteristics of the children according to whether they had at least one urinary tract admission during follow-up.

Characteristics	Children with at least one urinary tract admission		Predictors of urinary tract admission?	
	No	Yes	Multivariable HR	*p*-value
	*N* = 20,865 (98.2%)	*N* = 374 (1.8%)		
	Number (%)	Number (%)	(95% CI)^†^	
**Sex**				
Male	10,737 (98.5)	162 (1.5)	1.00	
Female	10,128 (97.9)	212 (2.1)	1.39 (1.14–1.71)	0.001
**Maternal age**				
<25	6,077 (97.8)	134 (2.2)	1.39 (1.00–1.94)	0.053
25–34	11,565 (98.4)	191 (1.6)	1.00	-
35+	3,223 (98.5)	49 (1.5)	1.07 (0.78–1.47)	0.665
Townsend score*				
1	3,493 (98.4)	56 (1.6)	1.00	-
2	3,305 (98.5)	49 (1.5)	0.91 (0.62–1.34)	0.633
3	4,031 (98.0)	81 (2.0)	1.19 (0.85–1.68)	0.312
4	4,726 (98.3)	83 (1.7)	1.00 (0.71–1.46)	0.996
5	5,158 (98.0)	103 (2.0)	1.12 (0.80–1.56)	0.513
Premature**				
No	19,721 (98.3)	331 (1.7)	1.00	-
Yes	1,091 (96.4)	41 (3.6)	2.29 (1.65–3.16)	<0.001

*Townsend deprivation score: 1 = least deprived, 5 = most deprived; missing data for 154 (0.7%).

**Prematurity: premature = <37 weeks gestation; missing data for 55 (0.3%).

^†^Multivariable models include all variables in the table; missing values for Townsend score and prematurity imputed using multiple imputation.

Abbreviations: CI, confidence interval; HR, hazard ratio.

Of the 21,101 children with no RPD at the FAS, 20,534 (97.3%) had no further investigations and 567 (2.7%) did (see Fig 2). Most of the 567 had no evidence of RPD at the later investigations (*n* = 523, 92.2%). In the group with no RPD at the FAS and no evidence of later dilatation or later investigations (*n* = 21,057), there were 333 children with at least one urinary tract hospital admission (1.6%). Most of these had only one admission (86.8%). The median age at first admission was 6 months (interquartile range [IQR] 2–14), and the commonest code linked to the first and all admissions was urinary tract infection (UTI; ICD-10 codes N39.0, N39.1, or P39.3).

A total of 26 of the 44 children (59.1%) with no RPD at the FAS but evidence of dilatation at later investigations had at least one urinary tract hospital admission, and most had more than one admission. The median age at first admission was three months (IQR 1–6), and the commonest code linked to the first and all admissions was hydronephrosis (ICD-10 code Q62.0).

Of the 138 children with RPD at the FAS, 88 (63.8%) had further investigations. A total of 59 of the 88 had no evidence of dilatation at the later investigations, and there were fewer than five hospital admissions in this group. No further investigations were found for 50 of the 138, and there were also fewer than five hospital admissions in this group. As described above, multiple imputation was used to account for the missing data on later dilatation in these children.

A total of 29 children with RPD at the FAS had evidence of later dilatation; 11 of these (37.9%) had at least one hospital admission for a urinary tract cause, and most had more than one admission. The median age at first admission was two months (IQR 0–15), and the commonest code linked to the first and all admissions was hydronephrosis (ICD-10 code Q62.0). Further details on the characteristics of first and all admissions for all groups are summarised in the supporting information (S2 Table and S3 Table).

The risk of at least one hospital admission was seven times greater in those with RPD (*n* = 21,101) compared with those without (*n* = 138, conditional HR [cHR] 7.23, 95% CI 4.31–12.15, *p* < 0.001, Table 3). Table 3 also shows the hazard ratios when the data were further stratified according to whether there was evidence of dilatation in later radiological investigations. Compared to those with no RPD and no later dilatation, the risk of urinary tract hospital admissions was higher in children with no RPD but dilatation detected later (cHR 62.06, 95% CI 41.10–93.71, *p* < 0.001). It was also higher in children with RPD and later dilatation (cHR 25.13, 95% CI 13.26–47.64, *p* < 0.001), but not in children with RPD and no later dilatation (cHR 2.16, 95% CI 0.69–6.75, *p* = 0.185). We also did not detect a difference at the 5% level in hospital admission rates between children with unilateral and bilateral RPD (Table 4). However, compared with children with no RPD and no later dilatation, there was evidence that children with bilateral RPD but no evidence of later dilatation had a higher risk of hospital admission than children with no RPD and no later dilatation (cHR 4.77, 95% CI 1.17–19.47, *p* = 0.029).

**Table 3:** HRs for time to first urinary tract hospital admission by RPD status at anomaly scan.

Time to first urinary tract hospital admission by RPD status (*n* = 21,239)				
	Univariate HR (95% CI)	*p*-value	Multivariable HR (95% CI)^‡^	*p*-value
**According to the presence of RPD at the anomaly scan**				
No RPD at anomaly scan	1.00	-	1.00	-
RPD at anomaly scan	6.91 (4.12–11.58)	<0.001	7.23 (4.31–12.15)	<0.001
**According to the presence of RPD at the anomaly scan and whether there is evidence of dilatation**^**†**^ **at later investigations**				
No RPD and no evidence of dilatation after the anomaly scan	1.00	-	1.00	-
No RPD and evidence of dilatation after the anomaly scan	61.99 (41.54–92.49)	<0.001	62.06 (41.10–93.71)	<0.001
RPD and no evidence of dilatation after the anomaly scan	2.04 (0.65–6.36)	0.219	2.16 (0.69–6.75)	0.185
RPD and evidence of dilatation after the anomaly scan	23.95 (12.70–45.15)	<0.001	25.13 (13.26–47.64)	<0.001

^†^Dilatation = evidence of dilatation of ≥7.1 mm later in pregnancy and/or evidence of dilatation of ≥7.1 mm postpartum.

^‡^Multivariable model also includes child sex, maternal age, Townsend score, gestational age at birth (multivariable model a better fit, likelihood ratio test *p* < 0.0001 in both cases).

Abbreviations: CI, confidence interval; HR, hazard ratio; RPD, renal pelvis dilatation.

**Table 4:** HRs for time to first urinary tract hospital admission with unilateral or bilateral RPD.

Time to first urinary tract hospital admission by RPD status (*n* = 21,239)				
	Univariate HR (95% CI)	*p*-value	Multivariable HR (95% CI)^‡^	*p*-value
**According to the presence of RPD at the anomaly scan**				
No RPD at anomaly scan	1.00	-	1.00	-
Unilateral RPD at anomaly scan	5.42 (2.69–10.92)	<0.001	5.54 (2.75–11.18)	<0.001
Bilateral RPD at anomaly scan	10.06 (4.76–21.25)	<0.001	11.17 (5.26–23.72)	< 0.001
**According to the presence of RPD at the anomaly scan and whether there is evidence of dilatation**^**†**^ **at later investigations**				
No RPD and no evidence of dilatation after the anomaly scan	1.00	-	1.00	-
No RPD and evidence of dilatation after the anomaly scan	61.99 (41.54–92.49)	<0.001	62.00 (41.06–93.63)	<0.001
Unilateral RPD and no evidence of dilatation after the anomaly scan	1.01 (0.14–7.22)	0.987	1.06 (0.15–7.56)	0.950
Bilateral RPD and no evidence of dilatation after the anomaly scan	4.37 (1.07–17.76)	0.039	4.77 (1.17–19.47)	0.029
Unilateral RPD and evidence of dilatation after the anomaly scan	22.06 (10.02–48.56)	<0.001	21.96 (9.91–48.67)	<0.001
Bilateral RPD and evidence of dilatation after the anomaly scan	27.45 (10.24–73.53)	<0.001	31.87 (11.72–86.68)	<0.001

†Dilatation = evidence of dilatation of ≥7.1 mm later in pregnancy and/or evidence of dilatation of ≥7.1 mm postpartum.

^‡^Multivariable model also includes child gender, maternal age, Townsend score, gestational age at birth (multivariable model a better fit, likelihood ratio test *p* < 0.0001 in both cases).

Abbreviations: CI, confidence interval; HR, hazard ratio; RPD, renal pelvis dilatation.

The results of sensitivity analyses are presented in the supporting information (S4 Table and S5 Table). When evidence of dilatation in the hospital admission records was included in the definition of the exposure subgroups, the HRs for children with evidence of later dilatation moved further away from one, whilst the estimate for children with RPD but no later dilatation moved closer to one. Accounting for multiple admissions increased the HRs for children with evidence of later dilatation, as these children were more likely to have multiple admissions, but did not change the conclusion for children with RPD but no later dilatation.

## Discussion

In this study, mild-to-moderate RPD was identified in 7.6 per 1,000 singleton pregnancies at the fetal anomaly ultrasound scan. In most children, this dilatation did not persist, and hospital admission rates for urinary tract problems in those children were similar to those in children with no RPD and no later dilatation. Persistent dilatation in later pregnancy and/or postpartum was rare, but children with this finding had a higher risk of hospital admission for a urinary tract cause before the age of three years. Children whose dilatation was identified later in pregnancy or postpartum had the highest risk of hospital admission.

### Strengths

This was a large population-based study in a cohort that was representative of all pregnant women in Wales. The stringent QA process was a strength of the study, with scan images reviewed by an expert panel to confirm that all cases of RPD conformed to the study definition. Linkage to routinely collected healthcare records was possible for 97% of women and children, ensuring that few were lost to follow-up. Unlike previous studies, we could compare outcomes in children with and without RPD. We used hospital admissions for urinary tract causes (including operations but not investigations) as a proxy for significant morbidity.

### Limitations

The prevalence of RPD in this study was similar to estimates in previous studies. However, the absolute number of cases was small (*n* = 138), and these numbers reduced further when the data were stratified by the presence or absence of later dilatation. The estimates from the study must therefore be interpreted with caution. Although the small numbers mean that the CIs around the point estimates are wide, the effect sizes for the associations examined are large, suggesting that the sample size for examining these associations is adequate.

The SAIL Databank only includes healthcare information from Wales, which means that we were unable to access information on pregnancy outcomes that occurred in facilities outside of Wales or on mothers and children who ordinarily received their healthcare outside of Wales (for example, because they live along the border with England). The distribution of pregnancy outcomes, congenital anomalies, and premature deliveries in the cohort included in our analysis was comparable with those in published data for Wales [26], suggesting that they were representative of the general obstetric population in Wales.

We used routinely available healthcare records on hospital admissions to capture data on outcomes in this study, as these are available for the whole population in Wales. They are also an indicator of clinically significant morbidity. However, we acknowledge that this means that we have not included less severe outcomes in our analysis, such as UTIs managed in the community or in outpatients, underestimating the total burden of these outcomes in the general population. It is also possible that provider practice may explain some of the increased risk in admissions seen with persistent dilatation, as they may be more likely to admit a child with a known kidney abnormality for treatment and observation. Without access to community or outpatient data, we cannot assess the effect of this potential bias on the estimates obtained.

Obtaining records of radiological investigations later in pregnancy and postpartum was challenging. Although guidelines state that all cases of RPD should receive follow-up investigations, we found later tests for only 88 of the 138 children with RPD (64%). This does not mean that they did not have later investigations, but that we were unable to find a record of these. Previous studies have restricted their samples to children with complete data on later pregnancy or postpartum scans (for example, see [34]), but this leads to an incomplete picture of the natural history of RPD, especially as the characteristics of the excluded sample are usually unreported. We were able to identify additional cases of ‘hydronephrosis’ from hospital admission codes and use this information in a sensitivity analysis to reclassify some children who had no data on later radiological investigations. This strengthened our conclusions, as admission rates for children without persistent dilatation reduced in this analysis.

When information from later radiological investigations was available, it was clear that the number, timing, and type of test was not consistent between children and that different terminology and measurement thresholds were used to identify dilatation in the radiological reports. Our findings suggest that regular training, clear reporting protocols, and frequent audits to monitor follow-up rates and maintain reporting standards are needed if the management of RPD is to be standardised.

### Implications for further research

Our results are consistent with findings from previous case series that have used repeated prenatal measurement of dilatation to predict which children require postnatal follow-up [35]. However, there were some additional questions that we were unable to answer in this study. For example, RPD was defined as an AP diameter of between 5.0 and 9.9 mm at the FAS, in accordance with the definition used by the antenatal screening programme in Wales when the data were collected [25]. Other classification systems [12] have further defined dilatation at the FAS as ‘mild’ (<7.0 mm) and ‘moderate’ (7.0 to ≤10 mm), and some screening programmes recommend that follow-up is only conducted when an RPD of ≥7.0 mm is identified at the FAS [8]. We could not assess whether there was a difference in hospital admissions between children with ‘mild’ or ‘moderate’ dilatation at the FAS, because the scan data collected for the study only contained information on whether RPD was present or not (but no measurements). Further studies are needed to document the natural history of dilatation and outcomes in children according to the actual measurement of the AP diameter at the FAS. We were also unable to draw firm conclusions about other predictors of poorer outcomes, such as unilateral compared with bilateral dilatation or the gender of the infant, because of sample size constraints. A larger study is needed to compare these subgroups. However, there was some evidence that children with bilateral RPD had a higher risk of hospital admissions even when there was no later evidence of dilatation, which is consistent with how bilateral RPD is currently managed in practice in Wales.

Two retrospective case series of patients known to specialist services have suggested that including other parameters (such as calyceal dilatation, renal parenchymal thickness, renal parenchymal appearance, bladder abnormalities, ureteral abnormalities, and oligohydramnios) in a formalised classification system is helpful in predicting which cases of dilatation will resolve spontaneously and which children require later surgery [36,37]. Further research is needed to fully understand the best combination of factors for predicting later pathology [9], and therefore allow for the development of care pathways with fewer follow-up visits or investigations for children identified as low risk [34]. Small studies have also examined different methods of visualising fetal urinary tract pathology (for example, conventional versus three-dimensional ultrasound) [38] and the use of serum and urinary biomarkers in the postpartum period to predict the risk of obstruction or impairment of renal function [39], but this research is currently inconclusive.

We also found that there were children who did not have RPD at the FAS (either 5.0 to 9.9 mm or ≥10 mm) who were found to have dilatation at later investigations. This is consistent with previous findings that not all cases of congenital dilatation are identified at the FAS [12]. In our study, this was a small group (44 of 21,101, 0.2%), but they had the highest rates of hospital admissions overall. As we did not have access to the full report from the FAS or later medical notes, we do not know why further investigations were conducted for these children. However, their hospital admission codes suggested that they had multiple congenital renal abnormalities and required more renal surgical procedures than children in the other groups. It is therefore likely that these children had other anomalies identified at the FAS, which led to the follow-up investigations and the repeated hospital admissions from an early age.

### Implications for clinical practice

Clear protocols for reporting and further investigation and management of RPD are being developed (for example, [10]), and regular audits are needed to ensure that these are followed. The effectiveness of continuous prophylactic antibiotics in the prevention of UTI in children with prenatally detected RPD is unclear, with evidence based on observational studies. The first trials of prophylactic antibiotics for antenatal hydronephrosis with postnatally diagnosed vesicoureteral reflux are under way (ClinicalTrials.gov Identifier: NCT01140516). Postnatal infections still occur despite prophylaxis [40], and antibiotic resistance is higher with increased use [41]. Studies have found an increased risk of UTI with severe prenatal dilatation in females, uncircumcised males, and with specific postnatal diagnoses (ureteral dilatation, vesicoureteral reflux, or vesico-ureteric junction obstruction) [42,43]. We did not have access to labour ward, outpatient, or general practitioner records in this study and, as such, did not have information on which children were given antibiotics after birth. Although we are therefore unable to comment on the role of antibiotics in preventing UTI, the main cause of admission in our study was for UTIs and our results support the need for close postnatal follow-up of children with persistent dilatation.

### Conclusion

RPD at the FAS is an important finding because it identifies fetuses who require later investigations. When these investigations are normal, we do not yet have sufficient evidence to tell whether there is an increase in hospital admissions for urinary tract problems in childhood. When there is persistent dilatation, hospital admission rates in childhood are higher. These results can be used to improve counselling of parents. Although this was a large study in a representative population of pregnant women in Wales, obtaining records of radiological investigations after the FAS was challenging, and scans were not conducted or reported consistently. Clear protocols for reporting and further investigation of RPD are being developed [10], and regular audits are needed to ensure that these are followed. Further studies should examine whether other characteristics at the FAS could improve the detection of renal pathology during antenatal screening.

## Supporting information

S1 STROBE checklist (DOC)

S1 Table Codes used in the definition of hospital admissions. (DOCX)

S2 Table Characteristics of the first hospital admissions. (DOCX)

S3 Table Characteristics of all hospital admissions. (DOCX)

S4 Table HRs for time to first urinary tract hospital admission, also using dilatation identified during hospital admissions to inform the classification of the exposure groups. HR, hazard ratio.
(DOCX)

S5 Table HRs accounting for multiple admissions. HR, hazard ratio.
(DOCX)

S1 Renal Study Protocol (DOCX)

## Abbreviations

ALF: anonymised linking field
AP: anterior-posterior
CAKUT: congenital anomalies of the kidney and urinary tract
CARIS: Congenital Anomaly Register for Wales
cHR: conditional hazard ratio
CI: confidence interval
CKD: chronic kidney disease
FAS: fetal anomaly scan
HR: hazard ratio
ICD-10: International Statistical Classification of Diseases and Related Health Problems, 10th Revision
IQR: interquartile range
NHS: National Health Service
OPCS: Operating Procedure Codes
PEDW: Patient Episode Database for Wales
QA: quality assurance
RadIS2: Radiology Information Service 2
RPD: renal pelvis dilatation
SAIL: Secure Anonymised Information Linkage
UTI: urinary tract infection

## References

[1] Warady B, Chadha V. Chronic kidney disease in children: the global perspective. Pediatr Nephrol. 2007;22(12):1999–2009. 10.1007/s00467-006-0410-1 17310363 PMC2064944

[2] Couser W, Remuzzi G, Mendis S, Tonelli M. The contribution of chronic kidney disease to the global burden of major noncommunicable diseases. Kidney International. 2011;80(12):1258–70. 10.1038/ki.2011.368 21993585

[3] Watson A. Psychosocial support for children and families requiring renal replacement therapy. Pediatr Nephrol. 2014;29(7):1169–74. 10.1007/s00467-013-2582-9 23963710

[4] Song R, Yosypiv I. Genetics of congenital anomalies of the kidney and urinary tract. Pediatr Nephrol. 2011;26(3):353–64. 10.1007/s00467-010-1629-4 20798957

[5] Harambat J, Van Stralen K, Kim J, Tizard E. Epidemiology of chronic kidney disease in children. Pediatr Nephrol. 2012;27(3):363–73. 10.1007/s00467-011-1939-1 21713524 PMC3264851

[6] Nguyen H, Herndon C, Cooper C, Gatti J, Kirsch A, Kokorowski P, et al The Society for Fetal Urology consensus statement on the evaluation and management of antenatal hydronephrosis. J Pediatr Urol. 2010;6(3):212–31. 10.1016/j.jpurol.2010.02.205 20399145

[7] Nef S, Neuhaus T, Spartà G, Weitz M, Buder K, Wisser J, et al Outcome after prenatal diagnosis of congenital anomalies of the kidney and urinary tract. Eur J Pediatr. 2016;175(5):667–76. 10.1007/s00431-015-2687-1 26805407

[8] Public Health England. NHS Fetal Anomaly Screening Programme Handbook. London: Public Health England, 8 2018.

[9] Chow J, Koning J, Back S, Nguyen H, Phelps A, Darge K. Classification of pediatric urinary tract dilation: the new language. Pediatr Radiol. 2017;47(9):1109–15. 10.1007/s00247-017-3883-0 28779200

[10] Antenatal Screening Wales. Ultrasound Observations Pathways: isolated echogenic bowel, isolated renal pelvis dilatation and isolated ventriculomegaly. Wales: Antenatal Screening Wales, 2018.

[11] Garne E, Loane M, Wellesley D, Barisic I, Group EW. Congenital hydronephrosis: prenatal diagnosis and epidemiology in Europe. J Pediatr Urol. 2009;5(1):47–52. 10.1016/j.jpurol.2008.08.010 18977697

[12] Nguyen H, Benson C, Bromley B, Campbell J, Chow J, Coleman B, et al Multidisciplinary consensus on the classification of prenatal and postnatal urinary tract dilation (UTD classification system). J Pediatr Urol. 2014;10(6):982–98. 10.1016/j.jpurol.2014.10.002 25435247

[13] Braga L, Ruzhynsky V, Pemberton J, Farrokhyar F, DeMaria J, Lorenzo A. Evaluating practice patterns in postnatal management of antenatal hydronephrosis: a national survey of Canadian pediatric urologists and nephrologists. Urology. 2014;83(4):909–14. 10.1016/j.urology.2013.10.054 24411215

[14] Zanetta V, Rosman B, Bromley B, Shipp T, Chow J, Campbell J, et al Variations in management of mild prenatal hydronephrosis among maternal-fetal medicine obstetricians, and pediatric urologists and radiologists. J Urology. 2012;88(5):1935–9. 10.1016/j.juro.2012.07.011 22999539

[15] Lee R, Cendron M, Kinnamon D, Nguyen H. Antenatal hydronephrosis as a predictor of postnatal outcome: a meta-analysis. Pediatrics. 2006;118(2):586–93. 10.1542/peds.2006-0120 16882811

[16] Braga L, Mijovic H, Farrokhyar F, Pemberton J, DeMaria J, Lorenzo A. Antibiotic prophylaxis for urinary tract infections in antenatal hydronephrosis. Pediatrics. 2013;131(1):e251–61. 10.1542/peds.2012-1870 23248229

[17] Harding L, Malone P, Wellesley D. Antenatal minimal hydronephrosis: is its follow‐up an unnecessary cause of concern? Prenatal Diag. 1999;19(8):701–5. 10451511 10.1002/(sici)1097-0223(199908)19:8<701::aid-pd621>3.0.co;2-5

[18] Walsh T, Hsieh S, Grady R, Mueller B. Antenatal hydronephrosis and the risk of pyelonephritis hospitalization during the first year of life. Urology. 2007;69(5):970–4. 10.1016/j.urology.2007.01.062 17482945

[19] Hall M, Piepsz A, Alexander M, Schulman C, Avni F. Insights into the pathogenesis and natural history of fetuses with renal pelvis dilatation. Eur Urol. 2005;48(2):207–14. 10.1016/j.eururo.2005.02.014 16005373

[20] Van Eerde A, Meutgeert M, De Jong T, Giltay J. Vesico‐ureteral reflux in children with prenatally detected hydronephrosis: a systematic review. Ultrasound Obst Gyn. 2007;29(4):463–9. 10.1002/uog.3975 17390310

[21] Hothi D, Wade A, Gilbert R, Winyard P. Mild Fetal Renal Pelvis Dilatation—Much Ado About Nothing? Clin J Am Soc Nephro. 2009;4(1):168–77. 10.2215/CJN.00810208 PMC2615699 18987299

[22] Swords K, Peters C. Neonatal and early infancy management of prenatally detected hydronephrosis. Arch Dis Child Fetal Neonatal Ed. 2015;100:F460–F4. 10.1136/archdischild-2014-306050 25605618

[23] Oliveira E, Oliveira M, Mak R. Evaluation and management of hydronephrosis in the neonate. Curr Opin Pediatr. 2016;28:195–201. 10.1097/MOP.0000000000000321 26807623

[24] Hurt L, Wright M, Brook F, Thomas S, Dunstan F, Fone D, et al The Welsh Study of Mothers and Babies: protocol for a population-based cohort study to investigate the clinical significance of defined ultrasound findings of uncertain significance. BMC Pregnancy Childb. 2014;14(1):164. 10.1186/1471-2393-14-164 PMC4029820 24884594

[25] Antenatal Screening Wales. Specific antenatal ultrasound findings: Guidelines for health professionals in Wales. Cardiff, UK: Antenatal Screening Wales, 2004.

[26] Hurt L, Wright M, Dunstan F, Thomas S, Brook F, Morris S, et al Prevalence of defined ultrasound findings of unknown significance at the second trimester fetal anomaly scan and their association with adverse pregnancy outcomes: the Welsh study of mothers and babies population‐based cohort. Prenatal Diag. 2016;36(1):40–8. 10.1002/pd.4708 26475362 PMC4949529

[27] World Health Organization. The ICD-10 classification of mental and behavioural disorders: Clinical descriptions and diagnostic guidelines. Geneva: World Health Organization, 1992.

[28] Health and Social Scre Information Centre. OPCS classification of interventions and procedures version 4.5. London: TSO (The Stationery Office), 2009.

[29] Ford D, Jones K, Verplancke J, Lyons R, John G, Brown G, et al The SAIL Databank: building a national architecture for e-health research and evaluation. BMC Health Serv Res. 2009;9(1):157. 19732426 10.1186/1472-6963-9-157 PMC2744675

[30] Lyons R, Jones K, John G, Brooks C, Verplancke J, Ford D, et al The SAIL databank: linking multiple health and social care datasets. BMC Med Inform Decis Mak. 2009;9(1):3. 19149883 10.1186/1472-6947-9-3 PMC2648953

[31] Yousaf S, Bonsall A. UK Townsend Deprivation Scores from 2011 census data. Colchester, UK: UK Data Service, 2017.

[32] White I, Royston P, Wood A. Multiple imputation using chained equations: issues and guidance for practice. Stat Med. 2011;30(4):377–99. 10.1002/sim.4067 21225900

[33] White I, Royston P. Imputing missing covariate values for the Cox model. Stat Med. 2009;28(15):1982–98. 10.1002/sim.3618 19452569 PMC2998703

[34] Zee R, Herndon C, Cooper C, Kim C, McKenna P, Khoury A, et al Time to resolution: A prospective evaluation from the Society for Fetal Urology hydronephrosis registry. J Pediatr Urol. 2017;13(3):316.e1–.e5. 28215834 10.1016/j.jpurol.2016.12.012

[35] Signorelli M, Cerri V, Taddei F, Groli C, Bianchi U. Prenatal diagnosis and management of mild fetal pyelectasis: implications for neonatal outcome and follow-up. Eur J Obstet Gyn R B. 2005;118(2):154–9. 10.1016/j.ejogrb.2004.04.023 15653195

[36] Hodhod A, Capolicchio J, Jednak R, El-Sherif E, El-Doray A, El-Sherbiny M. Evaluation of urinary tract dilation classification system for grading postnatal hydronephrosis. J Urology. 2016;195(3):725–30. 10.1016/j.juro.2015.10.089 26527513

[37] Kaspar C, Lo M, Bunchman T, Xiao N. The antenatal urinary tract dilation classification system accurately predicts severity of kidney and urinary tract abnormalities. J Pediatr Urol. 2017;13(5):485.e1–e7. 28499796 10.1016/j.jpurol.2017.03.020

[38] Duin L, Nijhuis J, Scherjon S, Vossen M, Willekes C. Comparison of conventional versus three-dimensional ultrasound in fetal renal pelvis measurement and their potential prediction of neonatal uropathies. J Matern-Fetal Neo M 2016;29(15):2493–8. 10.3109/14767058.2015.1090970 26430907

[39] Vemulakonda V, Yiee J, Wilcox D. Prenatal hydronephrosis: postnatal evaluation and management. Curr Urol Reps. 2014;15(8):430. 10.1007/s11934-014-0430-5 24927968

[40] Coelho G, Bouzada M, Lemos G, Pereira A, Lima B, Oliveira E. Risk factors for urinary tract infection in children with prenatal renal pelvic dilatation. J Urology. 2008;179(1):284–9. 10.1016/j.juro.2007.08.159 18001783

[41] Goossens H, Ferech M, Vander Stichele R, Elseviers M, ESAC Project Group. Outpatient antibiotic use in Europe and association with resistance: a cross-national database study. Lancet. 2005;365(9459):579–87. 10.1016/S0140-6736(05)17907-0 15708101

[42] Herz D, Merguerian P, McQuiston L. Continuous antibiotic prophylaxis reduces the risk of febrile UTI in children with asymptomatic antenatal hydronephrosis with either ureteral dilation, high-grade vesicoureteral reflux, or ureterovesical junction obstruction. J Pediatr Urol. 2014;10(4):650–4. 10.1016/j.jpurol.2014.06.009 25155409

[43] Braga L, Farrokhyar F, D'cruz J, Pemberton J, Lorenzo A. Risk factors for febrile urinary tract infection in children with prenatal hydronephrosis: a prospective study. J Urology. 2015;193(5S):1766–71. 10.1016/j.juro.2014.10.091 25813560

