# Supplementary material for: Mild-to-moderate renal pelvis dilatation identified during pregnancy and hospital admissions in childhood: An electronic birth cohort study in Wales, UK
Source: PLoS Med. 2019 Jul 30;16(7):e1002859. doi: 10.1371/journal.pmed.1002859 (PMC6667131; doi:10.1371/journal.pmed.1002859)
Supplement: S1 STROBE checklist — (DOC) [file pmed.1002859.s001.doc]

**STROBE Statement: Hospital admissions during childhood associated with mild-to-moderate renal pelvis dilatation identified during pregnancy: A prospective electronic birth cohort study**

|  | Item No | Recommendation | Page/line number in paper |
| --- | --- | --- | --- |
| **Title and abstract** | 1 | (*a*) Indicate the study’s design with a commonly used term in the title or the abstract | In title and Methods section of abstract |
|  |  | (*b*) Provide in the abstract an informative and balanced summary of what was done and what was found | In Abstract |
| Introduction | | |  |
| Background/rationale | 2 | Explain the scientific background and rationale for the investigation being reported | In first 3 paragraphs of Introduction |
| Objectives | 3 | State specific objectives, including any prespecified hypotheses | In 4^th^ paragraph of Introduction (1^st^ line) |
| Methods | | |  |
| Study design | 4 | Present key elements of study design early in the paper | In 1^st^-7^th^ paragraphs of Methods |
| Setting | 5 | Describe the setting, locations, and relevant dates, including periods of recruitment, exposure, follow-up, and data collection | In 1^st^-7^th^ paragraphs of Methods |
| Participants | 6 | (*a*) Give the eligibility criteria, and the sources and methods of selection of participants. Describe methods of follow-up | Inclusion and exclusion criteria in 3^rd^ paragraph of Methods  Follow-up methods in 7^th^ paragraph of Methods |
|  |  | (*b*) For matched studies, give matching criteria and number of exposed and unexposed | No matching |
| Variables | 7 | Clearly define all outcomes, exposures, predictors, potential confounders, and effect modifiers. Give diagnostic criteria, if applicable | In 4th-6th paragraphs of Methods & supporting information Table S1 |
| Data sources/ measurement | 8* | For each variable of interest, give sources of data and details of methods of assessment (measurement). Describe comparability of assessment methods if there is more than one group | In 4th-7th paragraphs of Methods |
| Bias | 9 | Describe any efforts to address potential sources of bias | In 7^th^ paragraph of Methods (low loss to follow-up due to good record linkage)  In 11^th^ paragraph of Methods (sensitivity analyses) |
| Study size | 10 | Explain how the study size was arrived at | In 8^th^ paragraph of Methods |
| Quantitative variables | 11 | Explain how quantitative variables were handled in the analyses. If applicable, describe which groupings were chosen and why | In 10^th^ paragraph of Methods |
| Statistical methods | 12 | (*a*) Describe all statistical methods, including those used to control for confounding | In 9^th^ and 11^th^ paragraph of Methods |
|  |  | (*b*) Describe any methods used to examine subgroups and interactions | In 5^th^ paragraph of Methods |
|  |  | (*c*) Explain how missing data were addressed | In 12^th^ paragraph of Methods |
|  |  | (*d*) If applicable, explain how loss to follow-up was addressed | In 3^rd^ paragraph of Methods |
|  |  | (*e*) Describe any sensitivity analyses | In 11^th^ paragraph of Methods |
| Results | | |  |
| Participants | 13* | (a) Report numbers of individuals at each stage of study—eg numbers potentially eligible, examined for eligibility, confirmed eligible, included in the study, completing follow-up, and analysed | Figure 1 (cohort flow diagram), described in 1^st^ paragraph of Results |
|  |  | (b) Give reasons for non-participation at each stage | Figure 1 (cohort flow diagram), described in 1^st^ paragraph of Results |
|  |  | (c) Consider use of a flow diagram | Figure 1 |
| Descriptive data | 14* | (a) Give characteristics of study participants (eg demographic, clinical, social) and information on exposures and potential confounders | Tables 1 and 2, described in 2^nd^ paragraph of Results |
|  |  | (b) Indicate number of participants with missing data for each variable of interest | Footnote to Table 1 |
|  |  | (c) Summarise follow-up time (eg, average and total amount) | In last line of 1^st^ paragraph of Results |
| Outcome data | 15* | Report numbers of outcome events or summary measures over time | Figure 2, described in 3^rd^-6^th^ paragraph of Results |
| Main results | 16 | (*a*) Give unadjusted estimates and, if applicable, confounder-adjusted estimates and their precision (eg, 95% confidence interval). Make clear which confounders were adjusted for and why they were included | Tables 3-4, described in 7^th^ paragraph of Results |
|  |  | (*b*) Report category boundaries when continuous variables were categorized | In 9^th^ paragraph of Methods |
|  |  | (*c*) If relevant, consider translating estimates of relative risk into absolute risk for a meaningful time period | Not applicable |
| Other analyses | 17 | Report other analyses done—eg analyses of subgroups and interactions, and sensitivity analyses | Supporting information Tables S4-S5, described in 9^th^ paragraph of Results |
| Discussion | | |  |
| Key results | 18 | Summarise key results with reference to study objectives | In 1^st^ paragraph of Discussion |
| Limitations | 19 | Discuss limitations of the study, taking into account sources of potential bias or imprecision. Discuss both direction and magnitude of any potential bias | In 3^rd^-7^th^ paragraphs of Discussion |
| Interpretation | 20 | Give a cautious overall interpretation of results considering objectives, limitations, multiplicity of analyses, results from similar studies, and other relevant evidence | In 8^th^-11^th^ paragraphs of Discussion |
| Generalisability | 21 | Discuss the generalisability (external validity) of the study results | In 4^th^ paragraph of Discussion |
| Other information | | |  |
| Funding | 22 | Give the source of funding and the role of the funders for the present study and, if applicable, for the original study on which the present article is based | Given on submission (including grant numbers) |

*Give information separately for exposed and unexposed groups.
