# Supplementary material for: Mild-to-moderate renal pelvis dilatation identified during pregnancy and hospital admissions in childhood: An electronic birth cohort study in Wales, UK
Source: PLoS Med. 2019 Jul 30;16(7):e1002859. doi: 10.1371/journal.pmed.1002859 (PMC6667131; doi:10.1371/journal.pmed.1002859)
Supplement: S1 Table — (DOCX) [file pmed.1002859.s002.docx]

**Table S1. Codes used in the definition of hospital admissions**

| **Admission cause** | **ICD-10 codes used to identify admissions** |
| --- | --- |
| Hydronephrosis | **Q62.0** (congenital hydronephrosis)  **N13.0** (with PUJO)  **N13.1** (with ureteral stricture)  **N13.2** (with renal and ureteral calculous obstruction)  **N13.3** (other and unspecified) |
| Other obstructive and reflux uropathy | N13.4 (hydroureter)N13.5 (kinking and stricture of ureter without hydronephrosis)N13.6 (pyonephrosis, conditions in N13.0-N13.5 with infection)N13.7 (vesicoureteral-reflux-associated uropathy)N13.8 (other obstructive and reflux uropathy)N13.9 (obstructive and reflux uropathy, unspecified) |
| Urinary tract infection | **N39.0** (site not specified)  **N39.1** (persistent proteinuria)  **P39.3** (neonatal UTI) |
| Nephritis | **N10** (acute interstitial nephritis, pyelitis, pyelonephritis)  **N11.0** (chronic interstitial nephritis, pyelitis, pyelonephritis)  **N11.1** (chronic obstructive pyelonephritis)  **N11.8** (other chronic tubulo-interstitial nephritis)  **N11.9** (chronic tubulo-interstitial nephritis, unspecified)  **N12** (tubulo-interstitial nephritis, not specified as acute or chronic) |
| Cystitis | **N30.0** (acute cystitis)  **N30.1** (interstitial cystitis, chronic)  **N30.2** (other chronic cystitis)  **N30.3** (trigonitis)  **N30.4** (irradiation cystitis)  **N30.8** (other cystitis, e.g. abscess of bladder)  **N30.9** (cystitis, unspecified) |
| Renal abscess | **N15.1** (renal and perinephric abscess) |
| Acute renal failure | **N17.0** (acute renal failure with tubular necrosis)  **N17.1** (acute renal failure with acute cortical necrosis)  **N17.2** (acute renal failure with medullary necrosis)  **N17.8** (other acute renal failure)  **N17.9** (acute renal failure, unspecified)  **N19** (unspecified renal failure)  **P96.0** (congenital renal failure) |
| Small or contracted kidney | **N26** (unspecified contracted kidney) N27.0 (small kidney, unilateral)N27.1 (small kidney, bilateral)N27.9 (small kidney, unspecified) |
| Other congenital renal conditions | **Q62.1** (atresia and stenosis of ureter)  **Q62.2** (congenital megaloureter)  **Q62.3** (other obstructive defects of renal pelvis and ureter)  **Q62.4** (agenesis of ureter)  **Q62.5** (duplication of ureter)  **Q62.6** (malposition of ureter)  **Q62.7** (congenital vesico-uretero-renal reflux)  **Q62.8** (other congenital malformations of ureter) Q64.2 (congenital posterior urethral valves)Q64.8 (other specified congenital malformations of urinary system)Q64.9 (congenital malformation of urinary system, unspecified) |
| Renal operations (OPCS-4 codes) | **M02** (total excision of kidney)  **M03** (partial excision of kidney)  **M08** (open operations of kidney)  **M10** (endoscopic operations of kidney)  **M13** (puncture of kidney)  **M16** (other kidney operations)  **M20** (replantation of ureter)  **M26** (therapeutic nephroscopic operations of kidney)  **M27** (therapeutic ureteroscopic operations of kidney)  **M32** (operations on ureteric orifice)  **M33** (percutaneous stent procedures)  **M38** (open drainage of bladder)  **M47** (catheterisation, only included if associated with another renal condition)  **M49** (other bladder operations)  **M76** (therapeutic endoscopic operations of urethra)  **M77** (examinations of urethra)  **M79** (other operations of urethra)  **M83** (other operations of urinary tract) |
| Other renal conditions | N15.8 (other specified renal tubulo-interstitial diseases)N15.9 (renal tubulo-interstitial disease, unspecified - Infection of kidney NOS) **N28.8** (other specified disorders of kidney and ureter - hypertrophy of kidney, megaloureter, nephroptosis, pyelitis/pyeloureteritis/ureteritis cystica, ureterocele)  **N28.9** (disorder of kidney and ureter, unspecified - nephropathy NOS, renal disease NOS) |
