# Supplementary material for: Mild-to-moderate renal pelvis dilatation identified during pregnancy and hospital admissions in childhood: An electronic birth cohort study in Wales, UK
Source: PLoS Med. 2019 Jul 30;16(7):e1002859. doi: 10.1371/journal.pmed.1002859 (PMC6667131; doi:10.1371/journal.pmed.1002859)
Supplement: S2 Table — (DOCX) [file pmed.1002859.s003.docx]

**Table S2. Characteristics of the first hospital admissions**

|  | **Total sample**  **(n = 21,239)** | **No RPD and no further dilatation**  **(n = 21,057)** | **RPD and no further dilatation**  **(n = 59)** | **RPD and no further investigations**  **(n = 50)** | **RPD and evidence of further dilatation**  **(n = 29)** | **No RPD and evidence of further dilatation**  **(n = 44)** |
| --- | --- | --- | --- | --- | --- | --- |
| **Total with at least 1 admission** | 374 | * | * | * | * | * |
| **Of those with an admission, percentage with only 1 admission** | 81.3% | 86.8% | n<5^*^ | n<5^*^ | 36.4% | 30.8% |
| **Median age at 1^st^ admission in months (IQR)** | 6 (2, 13) | 6 (2, 14) | n<5^*^ | n<5^*^ | 2 (0, 15) | 3 (1, 6) |
| **Percentage of 1^st^ admissions that are emergency** | 93.1% | 97.6% | n<5^*^ | n<5^*^ | 72.7% | 50.0% |
| **Median duration of 1^st^ admission in days (IQR)** | 1 (1, 3) | 2 (1,3) | n<5^*^ | n<5^*^ | 1 (1, 1) | 1 (1, 3) |
| **Most frequent condition codes in the 1^st^ admission^**^** | UTI (313)  Hydronephrosis (26)  Other congenital (26) | UTI (302)  Other infections (6)  Hydronephrosis (6)  Other congenital (11) | n<5^*^ | n<5^*^ | n<5^*^ | UTI (7)  Hydronephrosis (14)  Other congenital (11) |

^*^ There are less than 5 admissions in some of the sub-groups and including all totals would allow for the calculation of the numbers in those groups. Therefore, no data has been extracted. ^**^ Admission may have multiple urinary tract causes; the total number of children with other obstructive or reflux uropathies (including VUR) in the 1^st^ admission is <5.
