# Supplementary material for: Mild-to-moderate renal pelvis dilatation identified during pregnancy and hospital admissions in childhood: An electronic birth cohort study in Wales, UK
Source: PLoS Med. 2019 Jul 30;16(7):e1002859. doi: 10.1371/journal.pmed.1002859 (PMC6667131; doi:10.1371/journal.pmed.1002859)
Supplement: S3 Table — (DOCX) [file pmed.1002859.s004.docx]

**Table S3. Characteristics of all hospital admissions**

|  | **Total sample**  **(n = 21,239)** | **No RPD and no further dilatation**  **(n = 21,057)** | **RPD and no further dilatation**  **(n = 59)** | **RPD and no further investigations**  **(n = 50)** | **RPD and evidence of further dilatation**  **(n = 29)** | **No RPD and evidence of further dilatation**  **(n = 44)** |
| --- | --- | --- | --- | --- | --- | --- |
| **Total number of admissions** | 520 | * | * | * | * | * |
| **Median number of admissions (IQR)** | 1 (1, 1) | 1 (1, 1) | n<5^*^ | n<5^*^ | 2 (1, 6) | 2 (1, 4) |
| **Median age at admission in months (IQR)** | 7 (2, 17) | 7 (2, 17) | n<5^*^ | n<5^*^ | 8 (4, 21) | 6 (2, 15) |
| **Percentage of all admissions that are emergency** | 81.9% | 94.0% | n<5^*^ | n<5^*^ | 50.0% | 37.3% |
| **Median duration of all admissions in days (IQR)** | 1 (1, 3) | 1 (1, 3) | n<5^*^ | n<5^*^ | 1 (1,2) | 1 (1, 2) |
| **Most frequent condition codes in all admissions^**^** | * | UTI (349)  Other infections (7)  Hydronephrosis (12)  Other congenital (15)  Renal operations (13) | n<5^*^ | n<5^*^ | UTI (8)  Hydronephrosis (13)  Other obstructive or reflux uropathy (7)  Other congenital (16)  Renal operations (8) | UTI (12)  Hydronephrosis (42)  Other obstructive or reflux uropathy (13)  Other congenital (23)  Renal operations (14) |

^*^ There are less than 5 admissions in some of the sub-groups. Therefore, no data has been extracted; ^**^ Admission may have multiple urinary tract causes; the total number of children with other obstructive or reflux uropathies (including VUR) in all admission is <5 except for in the group with RPD but evidence of later dilatation.
