# Supplementary material for: Mild-to-moderate renal pelvis dilatation identified during pregnancy and hospital admissions in childhood: An electronic birth cohort study in Wales, UK
Source: PLoS Med. 2019 Jul 30;16(7):e1002859. doi: 10.1371/journal.pmed.1002859 (PMC6667131; doi:10.1371/journal.pmed.1002859)
Supplement: S4 Table — HR, hazard ratio. (DOCX) [file pmed.1002859.s005.docx]

**Table S4. Hospital admission rates and hazard ratios for time to first urinary tract hospital admission, also using dilatation identified during hospital admissions to inform the classification of the exposure groups**

| **Time to first urinary tract hospital admission by RPD status (n = 21,239)** | | |
| --- | --- | --- |
|  | **Univariate hazard ratio**  **(95% CI) ^**^** | **Multivariable hazard ratio**  **(95% CI) ^‡^** |
| **a. According to the presence of RPD at the anomaly scan** | | |
| No RPD at anomaly scan | 1.00 | 1.00 |
| RPD at anomaly scan | 6.91  (4.12, 11.58) | 7.23  (4.31, 12.15) |
| **b. According to the presence of RPD at the anomaly scan and whether there is evidence of dilatation† at later investigations** | | |
| No RPD and no evidence of dilatation after the anomaly scan | 1.00 | 1.00 |
| No RPD and evidence of dilatation after the anomaly scan | 73.48  (51.28, 105.29) | 76.69  (52.88, 111.21) |
| RPD and no evidence of dilatation after the anomaly scan | 0.73  (0.10, 5.17) | 0.77  (0.11, 5.48) |
| RPD and evidence of dilatation after the anomaly scan | 28.35  (15.62, 51.47) | 29.98  (16.42, 54.72) |

† Dilatation = evidence of dilatation of >=7.1mm later in pregnancy, and/or evidence of dilatation of >=7.1mm postpartum; ‡ Multivariable model also includes child gender, maternal age, Townsend score, gestational age at birth (multivariable model a better fit, likelihood ratio test p < 0.0001 in both cases)
