# Supplementary material for: Mild-to-moderate renal pelvis dilatation identified during pregnancy and hospital admissions in childhood: An electronic birth cohort study in Wales, UK
Source: PLoS Med. 2019 Jul 30;16(7):e1002859. doi: 10.1371/journal.pmed.1002859 (PMC6667131; doi:10.1371/journal.pmed.1002859)
Supplement: S5 Table — HR, hazard ratio. (DOCX) [file pmed.1002859.s006.docx]

**Table S5. Hazard ratios accounting for multiple admissions**

| **Hazard ratios for multiple hospital admission by RPD status (n = 21,239)** | | |
| --- | --- | --- |
|  | **Unadjusted hazard ratio**  **(95% CI)^**^** | **Multivariable hazard ratio**  **(95% CI)^‡^** |
| **a. According to the presence of RPD at the anomaly scan** | | |
| No RPD at anomaly scan | 1.00 | 1.00 |
| RPD at anomaly scan | 15.05  (7.46, 30.36) | 15.37  (7.80, 30.29) |
| **b. According to the presence of RPD at the anomaly scan and whether there is evidence of dilatation† at later investigations** | | |
| No RPD and no evidence of dilatation after the anomaly scan | 1.00 | 1.00 |
| No RPD and evidence of dilatation after the anomaly scan | 88.15  (59.40, 130.80) | 84.13  (54.64, 129.52) |
| RPD and no evidence of dilatation after the anomaly scan | 2.76  (0.77, 9.94) | 2.95  (0.83, 10.46) |
| RPD and evidence of dilatation after the anomaly scan | 55.58  (26.08, 118.43) | 56.85  (27.67, 116.84) |

Estimates from Anderson-Gill model to account for multiple admissions in some children (similar estimates obtained from marginal mean and PWP-TT models)

† Dilatation = evidence of dilatation of >=7.1mm later in pregnancy, and/or evidence of dilatation of >=7.1mm postpartum; ‡ Multivariable model also includes child gender, maternal age, Townsend score, gestational age at birth (Multivariable model a better fit, likelihood ratio test p < 0.0001 in both cases)
