# Supplementary material for: Mild-to-moderate renal pelvis dilatation identified during pregnancy and hospital admissions in childhood: An electronic birth cohort study in Wales, UK
Source: PLoS Med. 2019 Jul 30;16(7):e1002859. doi: 10.1371/journal.pmed.1002859 (PMC6667131; doi:10.1371/journal.pmed.1002859)
Supplement: S1 Renal Study Protocol — (DOCX) [file pmed.1002859.s007.docx]

**Welsh Study of Mothers and Babies: Prospective cohort study of renal disease in early childhood.**

**Study Protocol**

*Funder*: NISCHR (Ref: RFPPB-2012-1046)

**Aim**

We propose to use routinely collected health data to explore whether babies with a marker in one or both kidney/s (enlargement of the tubes that collect urine in the kidney, called pelvicalyceal dilatation) detected during pregnancy have more urinary tract infections or hospital admissions for kidney problems during childhood, compared to babies without a marker.

**Project summary**

Long term kidney disease is one of the most common illnesses of childhood causing lifelong health needs. Babies born with abnormalities in the kidney are at risk of long-term kidney disease that may require

treatment such as dialysis and kidney transplant as they grow older. Opportunities for early diagnosis and treatment of kidney disease may reduce the severity of illness over time. In some pregnancies the marker pelvicalyceal dilatation, is detected in the baby’s kidney at the antenatal ultrasound scan. The meaning of this marker for the future health of the baby (for example the risk of urine infections or hospital admissions for kidney problems) is not known. There are no consistent guidelines about how to treat babies with this condition and so the identification of this marker can lead to anxiety for families and additional tests during pregnancy and after birth. We were funded by NISCHR to study the meaning of markers of uncertain significance (Welsh Study of Mothers and Babies). The study has recruited 21,106 pregnant women, with consent to follow-up the babies in future studies. There are 150 babies in our study with the kidney marker. We did not find any associations with immediate birth outcomes such as stillbirth, premature birth or genetic problems.

We propose a prospective electronic cohort study in which we will record link the data on babies already

recruited to the Welsh Study of Mothers and Babies (WSMB) with routinely collected health care datasets in

Wales to obtain follow-up data on hospital admissions and diagnoses in primary care during childhood. We will

describe the natural history of pelvicalyceal dilatation (5-9.9mm – PCD) and compare the pattern of hospital

admissions, interventions and procedures due to renal causes and the incidence of urinary tract infection during childhood between:

1. Babies with no markers and no major congenital malformations

2. Babies with a renal marker (PCD, 5 - 9.9mm) on the 18-20 week antenatal ultrasound scan

3. Babies with congenital hydronephrosis (>10mm) on the 18-20 week antenatal ultrasound scan

The information obtained will play an important role in facilitating the development of appropriate clinical guidelines and care pathways during pregnancy and after birth, including the appropriate use of medical interventions and treatment. We plan to publish results in peer review journals.

**Background**

All pregnant women are offered a fetal anomaly scan at 18-20 weeks gestation to screen for structural

abnormalities in the fetus. In at least 5% of pregnancies, markers of unknown significance (Cardiac Echogenic

Foci, Choroid Plexus Cysts, Echogenic Bowel, Pelvicalyceal Dilatation (PCD), Mild Cerebral Ventriculomegaly,

Nuchal Thickening and Short Femur) are identified. The significance of these markers is unknown in relation to

the future health of the child, and hence subsequent management during pregnancy and after birth of the baby, as there have not been any follow-up studies that adequately address this. This has led to uncertainty among health care professionals as to whether to report these findings as part of the ultrasound scan report and how to explain the significance to women. As a result there is variation between hospitals in their clinical management^1^ and the information and follow-up care that is given to women and babies. The reporting of markers can cause unresolved maternal anxiety during pregnancy and leads to the offer of invasive procedures such as an amniocentesis and further scans for reassurance.^2^ The need for evidence-based policy on this topic led to calls for clinical research into the significance and implications of detection of all ultrasound ‘markers’ in unselected low-risk populations.^3^

We were funded by NISCHR to estimate the prevalence of individual markers at the mid-pregnancy fetal

anomaly scan, assess inter- and intra-sonographer variability in the detection of markers and set up a cohort of

pregnant women and babies that can be followed up in future studies to investigate associations between

specific markers and health outcomes (The Welsh Study of Mothers and Babies - WSMB).^4^ We successfully

recruited 21,106 pregnant women and obtained consent to follow-up of their babies in future studies by using the mother’s NHS number to access health information that is routinely collected about the mother and the baby. We found variation in the reporting of markers and set up an expert panel of radiologists and sonographers to validate the markers reported to this study.^5^ The focus of this proposed research is on PCD (pelvicalyceal dilatation 5-9.9mm) and associated health outcomes during childhood because of the clinical importance of chronic renal disease and the morbidity associated with acute episodes. Chronic kidney disease is one of the most common chronic illnesses of childhood.^6^ The majority of children with this condition will proceed to have kidney transplantation during childhood or will require lifelong medical care from adult nephrology services. The incidence and prevalence of chronic kidney disease has increased over the last five years and a proportion of patients present during infancy and childhood with congenital abnormalities.^6^ Opportunities for the early diagnosis and treatment of kidney disease may reduce the severity of illness over time.

In the WSMB, PCD (5-9.9mm) was reported in 192 babies; 94% (n=150) were confirmed by the study expert

panel. The validated prevalence of PCD (5 – 9.9mm) in our population study was 0.7% (95%CI 0.5%, 0.8%),

consistent with the lower end of the range reported in the literature (0.1% - 2.1%).^7^ This wide range is attributed to the majority of studies which were in selected populations with high-risk pregnancies and lack of consistency between studies in size and gestation used to define PCD. The significance of PCD (5–9.9mm) is unclear. There is debate about the size of PCD above which investigation is warranted due to the risk of vesico-ureteric reflux (VUR) and future urinary tract infections.^8-11^ There are no consistent data relating the size of PCD to outcomes during infancy and childhood so it is not possible to distinguish between cases with underlying pathology and those with no clinical consequences.^11-15^ Data from previous studies on natural history are usually for outcomes at birth^11,12,16-19^ and these are not comparable because of differences in the size and gestation used to define the marker between the studies. Consequently there are no consistent guidelines for perinatal and post-natal management and follow-up for this condition.

In Wales, following detection of PCD (5-9.9mm) at the 18–20 week ultrasound scan, a follow-up scan is offered

between 28 – 32 weeks, and if the dilatation is >7 - 8 mm (depending on local protocols) a scan is indicated in

the post-natal period. Congenital hydronephrosis (dilatation > 10mm) detected at the post-natal scan could

indicate VUR and/or obstruction, requiring treatment with prophylactic antibiotics or surgery. Preliminary analysis of data from the Congenital Anomaly Registry and Information Service for Wales (CARIS) showed that 58% of babies with PCD 5-9.9mm detected on antenatal ultrasound scan were followed-up at birth, and of these cases, 28% of babies had congenital hydronephrosis (PCD > 10mm). 80% of babies with congenital hydronephrosis detected on antenatal ultrasound scan (>10mm) were followed up at birth and 77% of these babies had this diagnosis confirmed on post-natal scan. Although congenital hydronephrosis is defined in Europe using the measurement of at least 10mm, local protocols indicate some hospitals use a measurement of at least 7mm to define and treat cases. There are no data on the degree of progression as determined by change in measurement between antenatal or post-natal scan or the influence of measurement error, and no information about the correlation between size of PCD and urinary tract morbidity, so it is not known whether or not the 10mm cut-off to define congenital hydronephrosis is appropriate or not.

One study set in the Netherlands investigated the incidence of urinary tract infections at age 5 – 6 years and

found no difference between babies with and without PCD (5-9.9mm).^20^ However the diagnosis of urinary tract

infection was solely by parental report with no medical diagnosis and so the question remains unanswered. No

study has compared hospital admissions for renal causes between babies with and without PCD.

The natural history and evidence for any association between PCD (5-10mm) and urinary tract outcomes in

childhood needs to be established in order to define clinical guidelines and requirements for subsequent management of pregnancy and postnatal follow-up for this condition.

**Study Design and Method**

**Study population and method**

This study will include babies born in Wales between 1st January 2009 and 31st December 2011 to mothers who consented to take part in the WSMB. All pregnant women receiving antenatal care in NHS Wales who had a mid pregnancy fetal anomaly scan provided by the NHS in Wales between 1st July 2008 – 31st March 2011 were eligible for recruitment to the study. Scan data were available for 74% of women in the study who had

singleton pregnancies (n=22,045). The maximum period of follow-up in this study will be five years, accounting for censoring by death or migration.

We will describe the natural history of PCD (5-9.9mm) and compare the pattern of hospital admissions for renal causes (using ICD10 codes N00–N99, Q60-Q64), interventions and procedures due to renal causes (using OPCS-4 codes), and the incidence of urinary tract infection (using Read codes) during childhood, in those with and without markers.

In order to carry out the analysis we will record link the babies in the WSMB to hospital admissions data and primary care data from general practices, with anonymised linkage of individual babies across the

datasets. This data linkage will be done by HIRU at Swansea University, using the SAIL databank.^21,22^ The data

contained within the databank are sensitive and HIRU have developed procedures to ensure the data is made

available for research whilst ensuring that individual confidentiality is protected, and have measures in place to ensure information governance standards are met.

**Data sources**

We will use the following datasets that are available for research use in the Secure Anonymised Information

Linkage (SAIL) databank available for research use from the Health Information Research Unit (HIRU) at

Swansea University.

1. Data for admissions to NHS hospitals is contained in the Patient Episode Database for Wales (PEDW),

produced by the collation of information from clinical coding of hospital admissions by NHS Wales Informatics

Service (NWIS) who are mandated to do this in Wales.

2. Primary care data for patients are available from participating general practices that contribute data to the

SAIL databank. These general practices provide records of primary care activity with Read codes defining the

nature of this activity. The SAIL databank currently contains records of primary care activity for approximately

50% of general practice in Wales, but it is anticipated that this coverage will increase before the start of this

project in response to on-going work by HIRU to achieve this.

3. Data on the presence of major or minor congenital anomalies is reported to a stringently and comprehensively

validated registry in Wales – CARIS.

4. Data on deaths and causes of death from the Office for National Statistics (ONS) Mortality files.

5. The Welsh Demographic Service for data on migration out of Wales.

**Outcome measures**

1. Proportion of children with any renal hospital admissions (defined by ICD-10 codes N00–N99, Q60-Q64

recorded in the primary or secondary coding position) and/or linked procedures (OPCS-4). We will define the

codes for renal ultrasound scan, micturating cystourethrogram (MCUG), Dimercaptosuccinic (DMSA) scan and

surgery for urinary tract obstruction using the Classification of interventions and procedures (OPCS-4) which is

used for the coding of operations, procedures and interventions performed in the NHS.

2. Incidence of urinary tract infections and repeat infections in children under age five diagnosed in primary care. These will be defined using Read codes for clinical activity and drug prescriptions relating to primary care

diagnosis, investigation and treatment of urinary tract infection. Read codes version 2.0 is available from the

Technology Reference data Update Distribution (TRUD) website, Department of Health, England.

3. Time to first hospital admission for renal conditions in children under age 5 defined by ICD10 codes N00 –

N99 and Q60 – 64 in the primary and secondary coding position. We will code the children who have migrated

out of Wales or who die so that we can take this censoring into account in the analysis.

4. Number of hospital admissions for renal conditions and urinary tract infections (as defined above) during

childhood, over a maximum follow-up period of five years.

**Statistical Analysis**

We will define three groups for analysis:

1. Babies with no markers and no major congenital malformations

2. Babies with a renal marker (PCD, 5 - 9.9mm) on the 18-20 week antenatal ultrasound scan

3. Babies with congenital hydronephrosis (>10mm) on the 18-20 week antenatal ultrasound scan

For groups 2 and 3 we will define three subgroups according to diagnosis at birth:

(a) no pathology (<7 mm)

(b) pelvicalyceal dilatation (7-9.9mm)

(c) confirmed congenital hydronephrosis (>10mm).

We will describe the natural history for babies in groups 2 and 3 including the detail of measurements of PCD at follow-up scans after birth using data from CARIS. We will describe the proportion of babies in sub-groups a, b and c, the proportion of children in these groups with any renal hospital admissions and/or linked procedures, and the incidence of urinary tract infections diagnosed in primary care.

For each baby we will define the length of follow-up and person-years at risk during the five-year study period,

accounting for censoring by death or migration. We will estimate and compare the proportion of hospital

admissions and the person-year incidence of urinary tract infections between groups 1, 2 and 3 according to

maternal age, parity, quintile of Townsend deprivation score quintile, gestational age, sex, and breast-feeding.

We will use time to event Cox’s regression analysis for the first admission to hospital for renal disease to obtain crude and adjusted hazard ratios for groups 2 and 3 as defined above compared to group 1; the adjustments will be for maternal age, parity, social deprivation, gestation, sex, birth-weight and breast-feeding.

We will model the number of urinary tract infections and hospital admissions for renal conditions separately using a negative binomial or Poisson model, depending on whether or not there is over-dispersion, allowing for the varying periods of time at risk for the children in the cohort, adjusting for the factors listed above.

**Sample size calculations**

Prelimiunary data from the PEDW dataset for the period 1998 to 2008 linked to 329,000 live births from WECC,

showed that there was a 1% chance of a renal admission before the first birthday, increasing to 2.8% by the

fourth birthday and 3.3% by the fifth birthday. The most common reasons for renal admission in children under 5 were urinary tract infection (ICD10 N39.0), non-specific renal condition (ICD10 39.9) and hydronephrosis (ICD10 N13.3). Admissions were more common in boys than girls, with a relative risk ratio of approximately 1.6. We will compare the rates of renal admissions between the 148 children with PCD and those without a marker. There will be 80% power for detecting a hazard ratio of at least 6.2 in admissions by the first birthday and for detecting a hazard ratio of 3.5 by the fourth birthday. Whilst these are large effect-sizes this study presents a unique opportunity to describe the natural histrory of PCD.

**Expected outputs of research**

The potential for maternal anxiety and difficulty in counselling women when markers are reported and the need for a consistent approach in the management of pelvicalyceal dilatation (PCD) is the principlal reason for this research. The impact of this work will be significant as information about the significance of renal ultrasound markers is not currently available. This work will address current gaps in the evidence-base about the health outcomes in infancy and childhood associated with renal pelvicalyceal dilatation. The findings from the study will further our understanding of the natural history of PCD and contribute significantly to the development of evidence-based clinical guidelines and care-pathways for the management of this condition. The findings will be used to provide accurate information for parents and avoid any unnecessary anxiety during pregnancy. Determination of the significance of this marker will also enable future cost-benefit analyses of reporting this marker and the utility of the marker in predicting health outcomes in children.

**Dissemination**

We will use standard dissemination strategies to make the study report available and present the findings from this study to relevant multi-disciplinary groups such as Antenatal Screening Wales, The National Specialist

Advisory Groups (NSAGs) to Welsh Government for Obstetrics and Paediatrics. We will also provide feedback to Health Boards and the Maternity Services Liaison Committee. We will also seek to disseminate study findings to pregnant women and parents of young children through our user representatives on the study steering group and the organisations they represent (e.g. The National Childbirth Trust) using appropriate websites and newsletters. In the Welsh Study for Mothers and Babies patient information leaflet we have told women that we will write to them about study findings in the future and we will do this by letter following the completion of the study. We will engage with the Children and Young People's Research Network and the Kidney Research Network (both NISCHR funded registered research groups). We will also present the findings of this study to academic audiences such as the British Maternal and Fetal Medicine Society, the British Association of Perinatal Medicine and The Royal College of Paediatricians. Each of these organisations have an annual conference in the United Kingdom and we will submit an abstract to present our work. We will also publish our work in peer-reviewed journals for dissemination to wider audiences.

*References*

1. Maclachlan N, Iskaros J, Chitty L, 2000. Ultrasound markers of fetal chromosomal abnormality: A survey of policies and practices in UK maternity ultrasound departments. Ultrasound in Obstetrics and Gynaecology, 15, 387-390.

2. Baille C, Mason G, 1997. The psychological impact of obstetric ultrasound scans and soft marker screening. Imaging, 9, 115-122.

3. NIHCE Clinical Guideline CG62: Antenatal care: Routine care for thehealthy pregnant woman. (March 2008) <http://www.nice.org.uk/cg62> (accessed 12.12.2012)

4. Welsh Study of Mothers and Babies. WAG/MRC Health Research Partnership. (Paranjothy PI, Fone, Dunstan co-PI) £199,323 April 2008- Dec 2012. Project ref: RFSHO7340

5. Welsh Study of Mothers and Babies. A population-based cohort study to investigate the clinical significance of Defined Ultrasound Findings of Uncertain Significance. Study report submitted to NISCHR December 2012

6. Kim JJ, Booth CJ, Waller S, et al. The demographic characteristics of children with chronic kidney disease stages 3-5 in South East England over a 5-year period. Arch Dis Child. Doi:10.1136/archdischild-2012-302400

7. Chudleigh PM. Chitty LS. Pembrey M. Campbell S. The association of aneuploidy and mild fetal pyelectasis in an unselected population: the results of a multicenter study. Ultrasound in Obstetrics & Gynecology. 17(3):197-202, 2001 Mar.

8. Ismaili K, Hall M, Donner C, et al. Results of systematic screeningfor minor degrees of fetal renal pelvis dilatation in an unselected population, Am J Obstet Gynecol 188: 242-246

9. Langer B. 2000. Fetal pyelectasis. Ultrasound Obstet Gynecol16:1-5

10.Ouzounian JG, Castro MA, Fresquez M, et al. Prognostic significance of antenatally detected fetal pyelectasis. Ultrasound Obstet Gynecol 7:424-428

11.Persutte WH, Koyle M, Lenke RR, et al. Mild pyelectasis ascertained with prenatal ultrasonography is pediatrically significant. Ultrasound Obstet Gynecol 10:12-18.

12.Adra AM, Mejides AA, Dennaoui MS, et al. Fetal pyelectasis: is it always physiologic? Am J Obstet Gynecol 173:1263-1266

13.Jawson MS, Dibble L, Puri S et al. 1999 Prospective study of outcome in antenatally diagnosed renal pelvis dilatation. Arch Dis Child Fetal Neonatal 80:F135-F138.

14.Kent A, Cox D, Downey P, et al. A study of mild fetal pyelectasia - outcome and proposed strategy for management. Prenat Diagn 20: 206-209

15.Langer B, Simeoni U, Montoya Y, et al. 1996. Antenatal diagnosis of upper unrinary tract dilatation by ultrasonography. Fetal Diagn Ther 11: 191-198

16.Sairam S, Al-Habib A, Sasson S, et al. Natural history of fetal hydronephrosis diagnosed on mid-trimester ultrasound. Ultrasound Obstet Gynecol 2001; 17:191-196

17.Feldman DM, DeCambre M, Kong E, et al. Evaluation and follow-up of fetal hydronephrosis. J Ultrasound Med 20: 1065-1069

18.Harding LJ, Malone PSJ, Wellesley DG. 1999. Antenatal minimal hydronephrosis: is its follow-up an unnecessary cause of concern? Prenat Diagn 19:701-705.

19.Morin L, Cendron M, Crombleholme TM, et al. Minimal hydronephrosis in the fetus: clinical significance and implications for management. J Urol 155: 2047-2049.

20.Damen-Elias HAM, Luijnenburg SE, Visser GHA, et al. Mild pyelectasis diagnosed by renal ultrasound is not a predictor of urinary tract morbidity in childhood. Prenat Diagn 2005; 25:1239-1247.

21.Ford DV, Jones KH, Verplancke JP, Lyons RA, John G, Brown G, et al. The SAIL Databank: building a national architecture for e-health research and evaluation. BMC Health Serv Res 2009;9:157.

22.Lyons RA, Jones KH, John G, Brooks CJ, Verplancke JP, Ford DV, et al. The SAIL databank: linking multiple health and social care datasets. BMC Medical Informatics and Decision Making 2009;9:3.
